# The lanthanum(III) molybdate(VI) La_4_Mo_7_O_27_

**DOI:** 10.1107/S1600536809026415

**Published:** 2009-07-11

**Authors:** Benjamin van der Wolf, Peter Held, Petra Becker

**Affiliations:** a Institute of Crystallography, University of Cologne, 50674 Cologne, Germany

## Abstract

Crystals of the ortho­rhom­bic phase La_4_Mo_7_O_27_ (lanthanum molybdenum oxide) were obtained from a non-stoichiometric melt in the pseudo-ternary system La_2_O_3_–MoO_3_–B_2_O_3_. In the crystal structure, distorted square-anti­prismatic [LaO_8_] and monocapped square-anti­prismatic [LaO_9_] polyhedra are connected *via* common edges and faces into chains along [010]. These chains are arranged in layers that alternate with layers of [MoO_4_] and [MoO_5_] polyhedra parallel to (001). In the molybdate layers, a distorted [MoO_5_] trigonal bipyramid is axially connected to two [MoO_4_] tetra­hedra, forming a [Mo_3_O_11_] unit.

## Related literature

The isoformular compounds Eu_4_Mo_7_O_27_ (Naruke & Yamase, 2001 ▶) and Gd_4_Mo_7_O_27_ (Naruke & Yamase, 2002 ▶) have a similar structure, but have monoclinic symmetry. Parameters needed to calculate bond-valence sums from bond lengths were taken from Brown & Altermatt (1985 ▶).

## Experimental

### 

#### Crystal data

La_4_Mo_7_O_27_
*M* *_r_* = 1659.22
Orthorhombic, 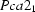

*a* = 14.1443 (14) Å
*b* = 7.2931 (4) Å
*c* = 22.9916 (13) Å
*V* = 2371.7 (3) Å^3^
*Z* = 4
Mo *K*α radiation
μ = 10.71 mm^−1^
*T* = 295 K
0.25 × 0.23 × 0.22 mm

#### Data collection

Nonius MACH3 diffractometer
Absorption correction: ψ scan (*MolEN*; Fair, 1990 ▶) *T*_min_ = 0.841, *T*_max_ = 0.999 (expected range = 0.080–0.095)
17773 measured reflections
7202 independent reflections
6225 reflections with *I* > 2σ(*I*)
*R*_int_ = 0.042
3 standard reflections every 100 reflections intensity decay: −4.1%

#### Refinement

*R*[*F* ^2^ > 2σ(*F* ^2^)] = 0.028
*wR*(*F* ^2^) = 0.076
*S* = 1.05
7202 reflections
345 parameters
1 restraint
Δρ_max_ = 2.27 e Å^−3^
Δρ_min_ = −1.35 e Å^−3^
Absolute structure: Flack (1983 ▶), 3515 Friedel pairs
Flack parameter: 0.039 (14)

### 

Data collection: *MACH3* (Enraf–Nonius, 1993 ▶); cell refinement: *MACH3*; data reduction: *MolEN* (Fair, 1990 ▶); program(s) used to solve structure: *SHELXS97* (Sheldrick, 2008 ▶); program(s) used to refine structure: *SHELXL97* (Sheldrick, 2008 ▶); molecular graphics: *DIAMOND* (Brandenburg, 2005 ▶); software used to prepare material for publication: *SHELXL97*.

## Supplementary Material

Crystal structure: contains datablocks I, global. DOI: 10.1107/S1600536809026415/wm2242sup1.cif

Structure factors: contains datablocks I. DOI: 10.1107/S1600536809026415/wm2242Isup2.hkl

Additional supplementary materials:  crystallographic information; 3D view; checkCIF report

## Figures and Tables

**Fig. 1.**
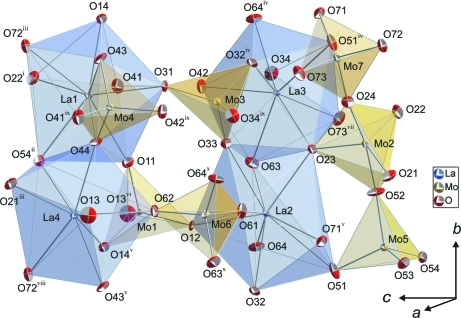
Fragment of the crystal structure of La4Mo7O27. Atoms are indicated as ellipsoids with probability regions of 50%. [Symmetry codes: (i) -x+1,-y,z+1/2; (ii) -x+1, -y-1, z+1/2; (iii) -x+3/2, y, z+1/2; (iv) x, y+1, z; (v) x, y-1, z; (vi) x-1/2, -y-1, z; (vii) x-1/2, -y, z; (viii) -x+3/2, y-1, z+1/2; (ix) x+1/2, -y, z; (x) x+1/2, -y-1, z.]

**Fig. 2.**
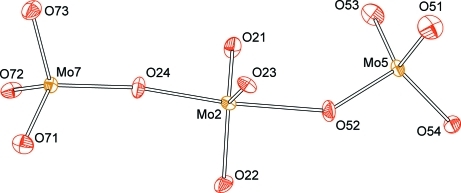
The trigonal-bipyramidal oxygen surrounding of Mo2. The two apical oxygen atoms are linked via corner-sharing to the tetrahedrally surrounded Mo5 and Mo7 atoms, forming a [Mo3O11] unit. Ellipsoid probability regions of 50% are given.

## supplementary crystallographic information

### Comment

The framework structure of La_4_Mo_7_O_27_ (Fig. 1) can be described as a layered arrangement of alternating [LaO_8_] and [LaO_9_] polyhedra and of [MoO_4_] and [MoO_5_] polyhedra parallel to (001). Perpendicular to this layered arrangement the [LaO_8_] and [LaO_9_] polyhedra are connected *via* edge- and face-sharing to form chains along [010]. The chains are interconnected *via* [MoO_4_] tetrahedra with an interchain distance of 1/2*a* = 7.072 Å. A similar layered arrangement of alternating [*RE*O_7_] and [*RE*O_8_] (*RE* = Eu, Gd) and of [MoO_4_] and [MoO_5_] polyhedra is present in the crystal strucures of Eu_4_Mo_7_O_27_ (Naruke & Yamase, 2001) and Gd_4_Mo_7_O_27_ (Naruke & Yamase, 2002). However, in the latter structures the rare earth oxygen polyhedra dimerize to [*RE*_2_O_12_] and [*RE*_2_O_13_] (*RE* = Eu, Gd) groups instead of forming chains. All four lanthanum atoms in La_4_Mo_7_O_27_ are found with a square-antiprismatic [LaO_8_] oxygen surrounding. In case of La1 and La3, this unit is monocapped to form a [LaO_9_] polyhedron, in case of La2 a more irregular but also monocapped (with O33) [LaO_9_] polyhedron may still be recognized, while La4 has a square-antiprismatic environment with two axial oxygen atoms O11 (La—O = 3.317 (6) Å) and O53 (La— O = 3.423 (7) Å) which we have not considered to be part of the La4 coordination polyhedron. Thereby, O53 is the only terminal oxygen atom connected to Mo5 only, but calculations of the bond valence sums (Brown & Altermatt, 1985) with 1.73 v.u. for the O53—Mo5 bond indicate no discrepancies and it can be assumed that the remaining bond charge is smeared out over farther atoms. The four molybdenum atoms Mo1, Mo4, Mo3, Mo6 are tetrahedrally surrounded by oxygen atoms resulting in [MoO_4_] tetrahedra with Mo—O distances ranging from 1.723 (7) to 1.805 (6) Å. These polyhedra are connected to [LaO_8_] or [LaO_9_] polyhedra *via* corner- or edge-sharing. This is also the case for Mo5 and Mo7, which are tetrahedrally surrounded and connected to [LaO_8_] or [LaO_9_] *via* corner-sharing only, but connected to Mo2 as well, which is fivefold surrounded to form a distorted trigonal bipyramid. Overall, this results in a [Mo_3_O_11_] unit (Fig. 2) with a corner-shared connection of the three equatorial oxygen atoms in the trigonal bipyramid to [LaO_8_] or [LaO_9_] polyhedra. In the trigonal bipyramid, the axial oxygen atom O52 has the longest Mo—O distance of 2.100 (6) Å in the structure, but bond valence sum calculations do suggest a trigonal-bipyramidal Mo2 surrounding with a resulting bond valence sum for the bonds Mo2—O21, O22, O23, O24 of 5.44 v.u., and when including also O52 of 6.04 v.u. In both Eu_4_Mo_7_O_27_ and Gd_4_Mo_7_O_27_ structures a similar [Mo_3_O_11_] unit is present, but here the two tetrahedrally surrounded molybdenum atoms are connected *via* one axial and one equatorial oxygen atom of the trigonal bipyramidal coordination polyhedron of the central molybdenum atom.

### Experimental

Single crystals of La_4_Mo_7_O_27_ of *ca* 0.01 mm^3^ in volume were synthesized by heating a homogenized powder mixture of La_2_O_3_ (99.99%, Chempur), H_3_BO_3_ (99.8%, Merck) and MoO_3_ (99.95%, Alfa Aesar) in a molar ratio of 0.16: 0.16: 0.68 in a covered platinum crucible in air atmosphere to 1023 K. After 95 h at this temperature the sample was quenched in air, washed with water, heated to 1100 K, cooled with 0.0013 K/min to 1093 K and quenched again in air. After a further similar heating-cooling cycle, colourless clear crystals of La_4_Mo_7_O_27_ were obtained and separated mechanically from the solidified melt.

### Refinement

In the final difference Fourier map the highest peak is 0.98 Å from atom O61 and the deepest hole is 0.78 Å from atom Mo7.

### Crystal data

**Table:**

La_4_Mo_7_O_27_	*F*(000) = 2952
*M**_r_* = 1659.22	*D*_x_ = 4.647 Mg m^−^^3^
Orthorhombic, *P**c**a*2_1_	Mo *K*α radiation, λ = 0.71073 Å
Hall symbol: P 2c -2ac	Cell parameters from 25 reflections
*a* = 14.1443 (14) Å	θ = 20.8–27.4°
*b* = 7.2931 (4) Å	µ = 10.71 mm^−^^1^
*c* = 22.9916 (13) Å	*T* = 295 K
*V* = 2371.7 (3) Å^3^	Prism, colourless
*Z* = 4	0.25 × 0.23 × 0.22 mm

### Data collection

**Table:**

Nonius MACH3 diffractometer	6225 reflections with *I* > 2σ(*I*)
Radiation source: fine-focus sealed X-ray tube	*R*_int_ = 0.042
graphite	θ_max_ = 30.4°, θ_min_ = 2.8°
ω/2θ scans	*h* = −20→20
Absorption correction: ψ scan (*MolEN*; Fair, 1990)	*k* = −10→10
*T*_min_ = 0.841, *T*_max_ = 0.999	*l* = −32→32
17773 measured reflections	3 standard reflections every 100 reflections
7202 independent reflections	intensity decay: −4.1%

### Refinement

**Table:**

Refinement on *F*^2^	Secondary atom site location: difference Fourier map
Least-squares matrix: full	*w* = 1/[σ^2^(*F*_o_^2^) + (0.04*P*)^2^ + 3.1563*P*] where *P* = (*F*_o_^2^ + 2*F*_c_^2^)/3
*R*[*F*^2^ > 2σ(*F*^2^)] = 0.028	(Δ/σ)_max_ = 0.001
*wR*(*F*^2^) = 0.076	Δρ_max_ = 2.27 e Å^−^^3^
*S* = 1.05	Δρ_min_ = −1.35 e Å^−^^3^
7202 reflections	Extinction correction: *SHELXL97* (Sheldrick, 2008), Fc^*^=kFc[1+0.001xFc^2^λ^3^/sin(2θ)]^-1/4^
345 parameters	Extinction coefficient: 0.00021 (2)
1 restraint	Absolute structure: Flack (1983), 3515 Friedel pairs
Primary atom site location: structure-invariant direct methods	Flack parameter: 0.039 (14)

### Special details

**Table:**

Geometry. All e.s.d.'s (except the e.s.d. in the dihedral angle between two l.s. planes) are estimated using the full covariance matrix. The cell e.s.d.'s are taken into account individually in the estimation of e.s.d.'s in distances, angles and torsion angles; correlations between e.s.d.'s in cell parameters are only used when they are defined by crystal symmetry. An approximate (isotropic) treatment of cell e.s.d.'s is used for estimating e.s.d.'s involving l.s. planes.
Refinement. Refinement of *F*^2^ against ALL reflections. The weighted *R*-factor *wR* and goodness of fit *S* are based on *F*^2^, conventional *R*-factors *R* are based on *F*, with *F* set to zero for negative *F*^2^. The threshold expression of *F*^2^ > σ(*F*^2^) is used only for calculating *R*-factors(gt) *etc*. and is not relevant to the choice of reflections for refinement. *R*-factors based on *F*^2^ are statistically about twice as large as those based on *F*, and *R*- factors based on ALL data will be even larger.

### Fractional atomic coordinates and isotropic or equivalent isotropic displacement parameters (Å^2^)

**Table:**

	*x*	*y*	*z*	*U*_iso_*/*U*_eq_	
La1	0.66087 (3)	0.01728 (6)	−0.111991 (17)	0.00665 (9)	
La2	0.53737 (3)	−0.51499 (6)	−0.346670 (18)	0.00663 (8)	
La3	0.40385 (3)	−0.00377 (6)	−0.348693 (18)	0.00607 (8)	
La4	0.79779 (3)	−0.49072 (6)	−0.105332 (18)	0.00796 (9)	
Mo1	0.56718 (5)	−0.50458 (8)	−0.17097 (3)	0.00648 (12)	
Mo2	0.52350 (5)	−0.21023 (9)	−0.48626 (3)	0.00797 (12)	
Mo3	0.63252 (5)	−0.01478 (9)	−0.28977 (3)	0.00647 (12)	
Mo4	0.88677 (5)	−0.00727 (8)	−0.16706 (3)	0.00596 (12)	
Mo5	0.42705 (5)	−0.67540 (9)	−0.50044 (3)	0.00924 (12)	
Mo6	0.80799 (5)	−0.48959 (8)	−0.29088 (3)	0.00678 (12)	
Mo7	0.69027 (5)	0.18663 (9)	−0.46606 (3)	0.00880 (12)	
O11	0.6097 (5)	−0.2827 (9)	−0.1607 (3)	0.0194 (13)	
O12	0.5618 (5)	−0.5645 (9)	−0.2443 (2)	0.0153 (12)	
O13	0.9529 (4)	−0.4730 (10)	−0.1422 (3)	0.0217 (15)	
O14	0.6456 (4)	0.3508 (8)	−0.1297 (2)	0.0112 (11)	
O21	0.6007 (4)	−0.3416 (9)	−0.5274 (3)	0.0157 (12)	
O22	0.4384 (4)	−0.0813 (9)	−0.5225 (3)	0.0158 (12)	
O23	0.5073 (4)	−0.2372 (8)	−0.4093 (2)	0.0120 (11)	
O24	0.6147 (5)	−0.0175 (8)	−0.4721 (3)	0.0155 (12)	
O31	0.6554 (5)	0.0575 (9)	−0.2186 (2)	0.0148 (12)	
O32	0.5662 (4)	−0.8518 (8)	−0.3325 (2)	0.0118 (11)	
O33	0.5583 (4)	−0.2059 (8)	−0.2937 (3)	0.0131 (12)	
O34	0.2400 (4)	0.0632 (9)	−0.3232 (3)	0.0169 (13)	
O41	0.4954 (5)	0.0377 (10)	−0.1345 (3)	0.0185 (13)	
O42	0.4018 (5)	0.0247 (10)	−0.2425 (3)	0.0198 (14)	
O43	0.8293 (5)	0.1988 (9)	−0.1478 (3)	0.0193 (14)	
O44	0.8030 (4)	−0.1691 (8)	−0.1377 (3)	0.0120 (11)	
O51	0.4290 (5)	−0.7789 (10)	−0.4302 (3)	0.0236 (15)	
O52	0.4298 (4)	−0.4333 (8)	−0.4899 (3)	0.0189 (13)	
O53	0.5258 (5)	−0.7338 (9)	−0.5387 (3)	0.0191 (13)	
O54	0.3242 (5)	−0.7323 (8)	−0.5428 (3)	0.0137 (12)	
O61	0.7039 (5)	−0.4836 (9)	−0.3311 (3)	0.0166 (13)	
O62	0.7826 (5)	−0.4752 (9)	−0.2169 (3)	0.0153 (13)	
O63	0.3730 (5)	−0.3124 (9)	−0.3092 (3)	0.0215 (14)	
O64	0.3901 (5)	−0.6789 (9)	−0.3162 (3)	0.0173 (13)	
O71	0.6315 (5)	0.3739 (8)	−0.4350 (3)	0.0164 (13)	
O72	0.7380 (4)	0.2446 (8)	−0.5355 (3)	0.0141 (12)	
O73	0.7859 (5)	0.1294 (9)	−0.4229 (3)	0.0189 (13)	

### Atomic displacement parameters (Å^2^)

**Table:**

	*U*^11^	*U*^22^	*U*^33^	*U*^12^	*U*^13^	*U*^23^
La1	0.00731 (18)	0.00518 (17)	0.00747 (19)	0.00098 (14)	0.00029 (15)	0.00006 (17)
La2	0.00777 (18)	0.00519 (18)	0.00694 (18)	0.00041 (14)	−0.00001 (15)	0.00120 (17)
La3	0.00763 (18)	0.00497 (18)	0.00560 (17)	−0.00037 (14)	0.00081 (15)	−0.00027 (15)
La4	0.0102 (2)	0.00603 (19)	0.00765 (19)	−0.00306 (15)	0.00111 (15)	−0.00116 (16)
Mo1	0.0075 (3)	0.0049 (3)	0.0070 (3)	0.0002 (2)	−0.0010 (2)	−0.0005 (2)
Mo2	0.0105 (3)	0.0084 (3)	0.0050 (3)	0.0013 (2)	0.0007 (2)	0.0003 (2)
Mo3	0.0072 (3)	0.0059 (3)	0.0063 (3)	0.0004 (2)	−0.0009 (2)	0.0008 (2)
Mo4	0.0063 (3)	0.0051 (3)	0.0065 (3)	−0.0005 (2)	0.0011 (2)	0.0000 (2)
Mo5	0.0138 (3)	0.0077 (3)	0.0062 (3)	−0.0019 (2)	−0.0017 (2)	−0.0001 (2)
Mo6	0.0069 (3)	0.0061 (3)	0.0073 (3)	−0.0007 (2)	0.0006 (2)	−0.0009 (2)
Mo7	0.0104 (3)	0.0103 (3)	0.0058 (2)	−0.0017 (2)	−0.0001 (2)	0.0017 (2)
O11	0.031 (4)	0.013 (3)	0.014 (3)	−0.004 (3)	0.003 (3)	−0.002 (2)
O12	0.030 (4)	0.012 (3)	0.004 (2)	−0.004 (3)	−0.003 (2)	−0.002 (2)
O13	0.006 (3)	0.031 (4)	0.028 (4)	−0.001 (3)	0.005 (3)	0.000 (3)
O14	0.009 (3)	0.011 (3)	0.014 (3)	0.000 (2)	−0.002 (2)	−0.002 (2)
O21	0.012 (3)	0.015 (3)	0.020 (3)	−0.001 (2)	0.008 (2)	−0.007 (2)
O22	0.012 (3)	0.021 (3)	0.015 (3)	−0.002 (2)	−0.006 (2)	0.004 (2)
O23	0.018 (3)	0.010 (3)	0.008 (3)	0.004 (2)	0.002 (2)	0.001 (2)
O24	0.014 (3)	0.015 (3)	0.018 (3)	−0.007 (2)	0.000 (3)	0.001 (2)
O31	0.024 (3)	0.015 (3)	0.006 (2)	−0.001 (3)	−0.006 (2)	0.000 (2)
O32	0.012 (3)	0.009 (3)	0.015 (3)	0.004 (2)	−0.005 (2)	0.002 (2)
O33	0.014 (3)	0.011 (3)	0.014 (3)	−0.006 (2)	−0.001 (2)	0.000 (2)
O34	0.008 (3)	0.023 (3)	0.020 (3)	0.000 (2)	0.003 (2)	−0.002 (3)
O41	0.012 (3)	0.025 (3)	0.018 (3)	0.005 (3)	−0.004 (2)	0.000 (3)
O42	0.028 (4)	0.023 (3)	0.009 (3)	0.001 (3)	0.001 (2)	0.001 (2)
O43	0.026 (4)	0.013 (3)	0.019 (3)	0.002 (3)	−0.004 (3)	−0.010 (2)
O44	0.011 (3)	0.008 (2)	0.017 (3)	0.001 (2)	0.005 (2)	−0.004 (2)
O51	0.029 (4)	0.031 (4)	0.011 (3)	0.000 (3)	0.001 (3)	0.010 (3)
O52	0.015 (3)	0.008 (3)	0.034 (4)	−0.003 (2)	0.005 (3)	−0.006 (3)
O53	0.025 (4)	0.016 (3)	0.015 (3)	0.004 (3)	0.003 (3)	−0.006 (2)
O54	0.015 (3)	0.014 (3)	0.011 (3)	−0.002 (2)	−0.006 (2)	0.000 (2)
O61	0.010 (3)	0.027 (4)	0.012 (3)	0.000 (3)	−0.005 (2)	−0.001 (2)
O62	0.022 (3)	0.018 (3)	0.006 (2)	0.004 (3)	0.002 (2)	0.000 (2)
O63	0.028 (4)	0.015 (3)	0.021 (3)	−0.004 (3)	0.003 (3)	0.007 (3)
O64	0.013 (3)	0.009 (3)	0.029 (3)	0.000 (2)	0.001 (3)	−0.004 (3)
O71	0.021 (3)	0.015 (3)	0.014 (3)	0.003 (2)	−0.002 (2)	−0.005 (2)
O72	0.012 (3)	0.021 (3)	0.010 (3)	0.003 (2)	0.001 (2)	0.003 (2)
O73	0.015 (3)	0.024 (3)	0.018 (3)	−0.003 (3)	−0.004 (2)	0.008 (3)

### Geometric parameters (Å, °)

**Table:**

La1—O41	2.402 (7)	La4—O14^v^	2.506 (6)
La1—O31	2.470 (6)	La4—O21^iii^	2.540 (6)
La1—O14	2.476 (6)	La4—O72^viii^	2.561 (6)
La1—O44	2.498 (6)	La4—O62	2.578 (6)
La1—O22^i^	2.535 (6)	La4—O54^ii^	2.773 (6)
La1—O11	2.562 (6)	La4—Mo1	3.5955 (9)
La1—O54^ii^	2.625 (6)	La4—La1^v^	4.0804 (6)
La1—O72^iii^	2.809 (6)	Mo1—O11	1.742 (6)
La1—O43	2.847 (7)	Mo1—O12	1.742 (6)
La1—Mo4	3.4416 (9)	Mo1—O13^vi^	1.754 (6)
La1—La4^iv^	4.0804 (6)	Mo1—O14^v^	1.801 (6)
La1—La4	4.1834 (7)	Mo2—O21	1.734 (6)
La2—O61	2.394 (6)	Mo2—O22	1.740 (6)
La2—O12	2.407 (6)	Mo2—O23	1.796 (6)
La2—O64	2.502 (7)	Mo2—O24	1.936 (6)
La2—O32	2.511 (6)	Mo2—O52	2.100 (6)
La2—O23	2.521 (6)	Mo3—O34^ix^	1.740 (6)
La2—O71^v^	2.559 (6)	Mo3—O33	1.747 (6)
La2—O33	2.579 (6)	Mo3—O31	1.749 (6)
La2—O63	2.886 (7)	Mo3—O32^iv^	1.805 (6)
La2—O51	3.121 (7)	Mo4—O41^ix^	1.723 (7)
La2—Mo6^vi^	3.4889 (9)	Mo4—O42^ix^	1.753 (6)
La2—La3^v^	4.0344 (6)	Mo4—O43	1.765 (6)
La2—La3	4.1797 (6)	Mo4—O44	1.804 (6)
La3—O34	2.439 (6)	Mo5—O53	1.704 (7)
La3—O42	2.450 (6)	Mo5—O52	1.782 (6)
La3—O63	2.466 (6)	Mo5—O51	1.784 (6)
La3—O64^iv^	2.492 (6)	Mo5—O54	1.799 (6)
La3—O51^iv^	2.515 (6)	Mo6—O61	1.739 (6)
La3—O73^vii^	2.557 (6)	Mo6—O62	1.741 (6)
La3—O32^iv^	2.577 (6)	Mo6—O63^x^	1.763 (7)
La3—O23	2.642 (6)	Mo6—O64^x^	1.788 (6)
La3—O33	2.923 (6)	Mo6—La2^x^	3.4889 (9)
La3—Mo3	3.5076 (9)	Mo7—O73	1.728 (6)
La3—La2^iv^	4.0344 (6)	Mo7—O71	1.752 (6)
La4—O13	2.356 (6)	Mo7—O72	1.784 (6)
La4—O44	2.462 (6)	Mo7—O24	1.838 (6)
La4—O43^v^	2.505 (6)		
			
O41—La1—O31	75.5 (2)	O51^iv^—La3—O32^iv^	72.6 (2)
O41—La1—O14	79.6 (2)	O73^vii^—La3—O32^iv^	146.4 (2)
O31—La1—O14	73.56 (19)	O34—La3—O23	141.4 (2)
O41—La1—O44	140.1 (2)	O42—La3—O23	125.9 (2)
O31—La1—O44	81.7 (2)	O63—La3—O23	72.8 (2)
O14—La1—O44	124.46 (19)	O64^iv^—La3—O23	144.5 (2)
O41—La1—O22^i^	67.9 (2)	O51^iv^—La3—O23	87.1 (2)
O31—La1—O22^i^	140.0 (2)	O73^vii^—La3—O23	77.2 (2)
O14—La1—O22^i^	84.5 (2)	O32^iv^—La3—O23	81.95 (19)
O44—La1—O22^i^	137.6 (2)	O34—La3—O33	135.00 (19)
O41—La1—O11	71.6 (2)	O42—La3—O33	67.7 (2)
O31—La1—O11	70.1 (2)	O63—La3—O33	60.8 (2)
O14—La1—O11	137.9 (2)	O64^iv^—La3—O33	114.10 (19)
O44—La1—O11	70.1 (2)	O51^iv^—La3—O33	123.0 (2)
O22^i^—La1—O11	110.8 (2)	O73^vii^—La3—O33	126.56 (19)
O41—La1—O54^ii^	104.9 (2)	O32^iv^—La3—O33	59.22 (17)
O31—La1—O54^ii^	134.2 (2)	O23—La3—O33	59.25 (17)
O14—La1—O54^ii^	152.20 (19)	O13—La4—O44	79.1 (2)
O44—La1—O54^ii^	69.36 (19)	O13—La4—O43^v^	75.1 (2)
O22^i^—La1—O54^ii^	72.5 (2)	O44—La4—O43^v^	137.6 (2)
O11—La1—O54^ii^	67.1 (2)	O13—La4—O14^v^	138.1 (2)
O41—La1—O72^iii^	126.5 (2)	O44—La4—O14^v^	113.4 (2)
O31—La1—O72^iii^	124.6 (2)	O43^v^—La4—O14^v^	69.5 (2)
O14—La1—O72^iii^	64.38 (18)	O13—La4—O21^iii^	72.8 (2)
O44—La1—O72^iii^	93.38 (19)	O44—La4—O21^iii^	77.8 (2)
O22^i^—La1—O72^iii^	70.44 (19)	O43^v^—La4—O21^iii^	124.1 (2)
O11—La1—O72^iii^	157.2 (2)	O14^v^—La4—O21^iii^	147.2 (2)
O54^ii^—La1—O72^iii^	92.70 (18)	O13—La4—O72^viii^	116.8 (2)
O41—La1—O43	136.4 (2)	O44—La4—O72^viii^	155.97 (19)
O31—La1—O43	71.6 (2)	O43^v^—La4—O72^viii^	66.3 (2)
O14—La1—O43	64.43 (19)	O14^v^—La4—O72^viii^	67.87 (19)
O44—La1—O43	60.74 (19)	O21^iii^—La4—O72^viii^	89.5 (2)
O22^i^—La1—O43	127.78 (19)	O13—La4—O62	73.5 (2)
O11—La1—O43	120.5 (2)	O44—La4—O62	70.1 (2)
O54^ii^—La1—O43	118.4 (2)	O43^v^—La4—O62	70.5 (2)
O72^iii^—La1—O43	58.68 (17)	O14^v^—La4—O62	74.1 (2)
O61—La2—O12	74.2 (2)	O21^iii^—La4—O62	136.9 (2)
O61—La2—O64	145.4 (2)	O72^viii^—La4—O62	129.9 (2)
O12—La2—O64	76.9 (2)	O13—La4—O54^ii^	137.2 (2)
O61—La2—O32	85.1 (2)	O44—La4—O54^ii^	67.43 (18)
O12—La2—O32	72.7 (2)	O43^v^—La4—O54^ii^	147.4 (2)
O64—La2—O32	68.4 (2)	O14^v^—La4—O54^ii^	81.49 (19)
O61—La2—O23	100.1 (2)	O21^iii^—La4—O54^ii^	74.64 (19)
O12—La2—O23	134.7 (2)	O72^viii^—La4—O54^ii^	89.67 (18)
O64—La2—O23	113.8 (2)	O62—La4—O54^ii^	115.98 (19)
O32—La2—O23	152.58 (19)	O11—Mo1—O12	112.3 (3)
O61—La2—O71^v^	68.7 (2)	O11—Mo1—O13^vi^	110.7 (3)
O12—La2—O71^v^	130.8 (2)	O12—Mo1—O13^vi^	107.5 (3)
O64—La2—O71^v^	120.3 (2)	O11—Mo1—O14^v^	105.1 (3)
O32—La2—O71^v^	73.1 (2)	O12—Mo1—O14^v^	112.9 (3)
O23—La2—O71^v^	83.65 (19)	O13^vi^—Mo1—O14^v^	108.4 (3)
O61—La2—O33	74.5 (2)	O21—Mo2—O22	118.2 (3)
O12—La2—O33	69.7 (2)	O21—Mo2—O23	123.9 (3)
O64—La2—O33	112.4 (2)	O22—Mo2—O23	116.3 (3)
O32—La2—O33	140.80 (18)	O21—Mo2—O24	94.2 (3)
O23—La2—O33	65.57 (19)	O22—Mo2—O24	98.6 (3)
O71^v^—La2—O33	126.2 (2)	O23—Mo2—O24	90.0 (3)
O61—La2—O63	134.4 (2)	O21—Mo2—O52	87.0 (3)
O12—La2—O63	84.3 (2)	O22—Mo2—O52	87.9 (3)
O64—La2—O63	59.4 (2)	O23—Mo2—O52	82.8 (3)
O32—La2—O63	126.34 (19)	O24—Mo2—O52	171.9 (3)
O23—La2—O63	67.86 (18)	O34^ix^—Mo3—O33	109.9 (3)
O71^v^—La2—O63	144.88 (19)	O34^ix^—Mo3—O31	108.2 (3)
O33—La2—O63	60.28 (19)	O33—Mo3—O31	113.6 (3)
O61—La2—O51	129.4 (2)	O34^ix^—Mo3—O32^iv^	110.3 (3)
O12—La2—O51	125.4 (2)	O33—Mo3—O32^iv^	100.7 (3)
O64—La2—O51	57.9 (2)	O31—Mo3—O32^iv^	114.0 (3)
O32—La2—O51	63.67 (18)	O41^ix^—Mo4—O42^ix^	108.2 (3)
O23—La2—O51	93.53 (18)	O41^ix^—Mo4—O43	114.2 (3)
O71^v^—La2—O51	64.7 (2)	O42^ix^—Mo4—O43	111.5 (3)
O33—La2—O51	152.36 (19)	O41^ix^—Mo4—O44	109.7 (3)
O63—La2—O51	96.0 (2)	O42^ix^—Mo4—O44	113.8 (3)
O34—La3—O42	74.5 (2)	O43—Mo4—O44	99.3 (3)
O34—La3—O63	85.8 (2)	O53—Mo5—O52	107.4 (3)
O42—La3—O63	73.0 (2)	O53—Mo5—O51	110.4 (3)
O34—La3—O64^iv^	70.3 (2)	O52—Mo5—O51	107.2 (3)
O42—La3—O64^iv^	67.6 (2)	O53—Mo5—O54	109.0 (3)
O63—La3—O64^iv^	138.0 (2)	O52—Mo5—O54	108.6 (3)
O34—La3—O51^iv^	100.5 (2)	O51—Mo5—O54	113.9 (3)
O42—La3—O51^iv^	133.5 (2)	O61—Mo6—O62	110.1 (3)
O63—La3—O51^iv^	153.4 (2)	O61—Mo6—O63^x^	109.6 (3)
O64^iv^—La3—O51^iv^	67.3 (2)	O62—Mo6—O63^x^	113.0 (3)
O34—La3—O73^vii^	67.2 (2)	O61—Mo6—O64^x^	111.1 (3)
O42—La3—O73^vii^	133.4 (2)	O62—Mo6—O64^x^	114.2 (3)
O63—La3—O73^vii^	78.7 (2)	O63^x^—Mo6—O64^x^	98.4 (3)
O64^iv^—La3—O73^vii^	119.3 (2)	O73—Mo7—O71	109.0 (3)
O51^iv^—La3—O73^vii^	80.2 (2)	O73—Mo7—O72	106.0 (3)
O34—La3—O32^iv^	136.5 (2)	O71—Mo7—O72	111.1 (3)
O42—La3—O32^iv^	80.2 (2)	O73—Mo7—O24	107.6 (3)
O63—La3—O32^iv^	119.8 (2)	O71—Mo7—O24	112.8 (3)
O64^iv^—La3—O32^iv^	67.5 (2)	O72—Mo7—O24	110.1 (3)

Symmetry codes: (i) −*x*+1, −*y*, *z*+1/2; (ii) −*x*+1, −*y*−1, *z*+1/2; (iii) −*x*+3/2, *y*, *z*+1/2; (iv) *x*, *y*+1, *z*; (v) *x*, *y*−1, *z*; (vi) *x*−1/2, −*y*−1, *z*; (vii) *x*−1/2, −*y*, *z*; (viii) −*x*+3/2, *y*−1, *z*+1/2; (ix) *x*+1/2, −*y*, *z*; (x) *x*+1/2, −*y*−1, *z*.
